# The associations of parity and maternal age with small-for-gestational-age, preterm, and neonatal and infant mortality: a meta-analysis

**DOI:** 10.1186/1471-2458-13-S3-S2

**Published:** 2013-09-17

**Authors:** Naoko Kozuki, Anne CC Lee, Mariangela F Silveira, Ayesha Sania, Joshua P Vogel, Linda Adair, Fernando Barros, Laura E Caulfield, Parul Christian, Wafaie Fawzi, Jean Humphrey, Lieven Huybregts, Aroonsri Mongkolchati, Robert Ntozini, David Osrin, Dominique Roberfroid, James Tielsch, Anjana Vaidya, Robert E Black, Joanne Katz

**Affiliations:** 1 Department of International Health, Johns Hopkins Bloomberg School of Public Health, 615 N. Wolfe St., Baltimore, MD 21205, USA; 2 Brigham and Women’s Hospital, 75 Francis Street, Boston, MA 02115, USA; 3 Programa de Pós-graduacao em Epidemiologia, Universidade Federal de Pelotas, Rua Marechal Deodoro 1160, 3o piso, Centro, CEP 96020-220, Pelotas, RS, Brazil; 4 Department of Epidemiology, Harvard School of Public Health, 677 Huntington Ave, Boston, MA 02115 USA; 5 School of Population Health, Faculty of Medicine, Dentistry and Health Sciences, University of Western Australia, 35 Stirling Highway Crawley WA 6009, Perth, Australia; 6 UNDP/UNFPA/UNICEF/WHO/Word Bank Special Programme of Research, Development and Research Training in Human Reproduction (HRP), Department of Reproductive Health and Research, World Health Organization, Geneva, Switzerland; 7 University of North Carolina School of Public Health, 135 Dauer Drive, Chapel Hill, NC 27599, USA; 8 Programa de Pós-graduação em Saúde e Comportamento, Univertsidade Católica de Pelotas, Félix da Cunha, 412, CEP 96010-000, Centro, Pelotas, RS, Brazil; 9 Departments of Nutrition, Epidemiology, and Global Health and Population, Harvard School of Public Health, 677 Huntington Ave, Boston, MA 02115 USA; 10 Zvitambo, No 1 Borrowdale Road, Borrowdale, Harare, Zimbabwe; 11 Department of Food Safety and Food Quality, Ghent University, Coupure Links 653, B – 9000, Ghent, Belgium; 12 Woman and Child Health Research Center, Department of Public Health, Institute of Tropical Medicine Nationalestraat 155, 2000 Antwerpen, Belgium; 13 ASEAN Institute for Health Development, Mahidol University, 999 Phuttamonthon 4 Rd, Salaya, Nakhon Pathom 73170, Thailand; 14 Institute for Global Health, UCL Institute of Child Health, 30 Guilford Street, London WC1N 1EH, UK

## Abstract

**Background:**

Previous studies have reported on adverse neonatal outcomes associated with parity and maternal age. Many of these studies have relied on cross-sectional data, from which drawing causal inference is complex. We explore the associations between parity/maternal age and adverse neonatal outcomes using data from cohort studies conducted in low- and middle-income countries (LMIC).

**Methods:**

Data from 14 cohort studies were included. Parity (nulliparous, parity 1-2, parity ≥3) and maternal age (<18 years, 18-<35 years, ≥35 years) categories were matched with each other to create exposure categories, with those who are parity 1-2 and age 18-<35 years as the reference. Outcomes included small-for-gestational-age (SGA), preterm, neonatal and infant mortality. Adjusted odds ratios (aOR) were calculated per study and meta-analyzed.

**Results:**

Nulliparous, age <18 year women, compared with women who were parity 1-2 and age 18-<35 years had the highest odds of SGA (pooled adjusted OR: 1.80), preterm (pooled aOR: 1.52), neonatal mortality (pooled aOR: 2.07), and infant mortality (pooled aOR: 1.49). Increased odds were also noted for SGA and neonatal mortality for nulliparous/age 18-<35 years, preterm, neonatal, and infant mortality for parity ≥3/age 18-<35 years, and preterm and neonatal mortality for parity ≥3/≥35 years.

**Conclusions:**

Nulliparous women <18 years of age have the highest odds of adverse neonatal outcomes. Family planning has traditionally been the least successful in addressing young age as a risk factor; a renewed focus must be placed on finding effective interventions that delay age at first birth. Higher odds of adverse outcomes are also seen among parity ≥3 / age ≥35 mothers, suggesting that reproductive health interventions need to address the entirety of a woman’s reproductive period.

**Funding:**

Funding was provided by the Bill & Melinda Gates Foundation (810-2054) by a grant to the US Fund for UNICEF to support the activities of the Child Health Epidemiology Reference Group.

## Introduction

Parity and maternal age have been shown to increase the risk of adverse neonatal outcomes, such as intrauterine growth restriction (IUGR), prematurity, and mortality [1-5]. Nulliparity may confer risk through complications during childbirth such as obstructed labor [6], whereas high parity has been linked to increased risk of hypertension, placenta previa, and uterine rupture [4]. Several studies have hypothesized that in young mothers, maternal-fetal competition for nutrients and/or the mother’s incomplete physical growth might contribute to adverse neonatal outcomes [7]. Older women experience an increase in the incidence of congenital abnormalities as well as maternal morbidities such as hypertension and gestational diabetes [8,9]. However, some literature has suggested that controlling for socioeconomic status heavily attenuates or eliminates associations of adolescence and of high parity with adverse outcomes [10,11].

Despite the abundance of existing literature on parity and maternal age as risk factors for adverse neonatal outcomes, methodological issues in many studies make it difficult to draw strong conclusions. Several studies have utilized cross-sectional data, often Demographic and Health Surveys (DHS) [12-14]. Cross-sectional studies cannot assess causality easily. Furthermore, information recorded at the time of interview, i.e. confounders like socioeconomic factors, may fail to reflect the true condition at the time of pregnancy and delivery. In addition, the quality of relevant variables may be poor in surveys. For example, gestational age data dependent on maternal recall are expected to be less accurate than data from prospective cohort studies where women of reproductive age are closely tracked and/or estimated gestational age is corroborated through clinical assessment.

Studies have also failed to examine the potential confounding effects of other reproductive health-related variables, socioeconomic status, or maternal nutrition. One systematic review found an association between nulliparity and SGA, but not prematurity; however, it failed to limit the studies included in the meta-analysis to those that controlled for maternal age [4]. Furthermore, the studies that do control for these confounders often fail to indicate whether the adjustment may have altered the associations [3], preventing us from understanding the biological or confounding mechanisms linking parity and maternal age to poor outcomes.

Categorizations and definitions of risk factors and outcomes described in the present literature have differed across studies. For example, the low-risk reference category for parity varies substantially across studies (i.e. birth orders 2-3 [4], 2-4 [15], and 2-5 [16]). Definitions of young age also differ, with cut-offs ranging from 16 to 18 [2], and some authors have evaluated age since first menarche. Definitions of outcomes also vary. Various proxies for IUGR have been used [17,18], the most common being small-for-gestational-age (SGA) defined as birthweight below the 10^th^ percentile of a gender-specific reference distribution of birthweight at a particular gestational age. Such variety in exposure and outcome definitions makes it difficult to compare results across studies.

An objective of the family planning section of this supplement is to derive the best estimate of associations between reproductive health-related maternal risk factors and adverse neonatal outcomes to include in the Lives Saved Tool (*LiST*). *LiST* is a software product that produces evidence-based estimates of changes in maternal and child mortality if a health intervention is scaled up in a particular country [19]. We used original data from prospective cohort studies to examine parity and maternal age as exposures for adverse neonatal and infant outcomes. Our aim was to apply standardized categorizations and definitions for the exposure and outcome variables across studies in order to obtain best estimates. We controlled for available confounders such as socioeconomic status and maternal nutrition to examine their effect on associations. The findings will help us understand if and how family planning programs may impact neonatal and infant survival, as well as better evaluate the potential mechanisms linking these risk factors to adverse outcomes.

## Methods

Our general approach was to identify individual prospective birth cohorts for which we conducted a standardized set of analyses, and then to meta-analyze the individual study associations. First, population-based, prospective cohort studies from LMIC with information on parity and maternal age, newborn birthweight (collected within the first 72 hours), and gestational age were identified from studies extracted for a separate analysis [Table 1]. Briefly, the separate analysis sought to estimate neonatal and infant mortality risk of SGA and preterm births. Datasets were identified through a literature review conducted in September 2009. We searched Medline and WHO regional databases to identify birth cohorts that contained relevant data including gestational age, birthweight, and vital status on newborns up to at least one month of life. Investigators of those studies were contacted and invited to contribute data or conduct analysis using a standardized analysis plan. Additional birth cohorts from ongoing or recently completed maternal-health studies were identified by word-of-mouth by members of the Child Health Epidemiology Reference Group (CHERG) SGA-Preterm Birth working group. More details are available in a separate publication [20]. Fourteen datasets were identified [21-35].

**Table 1:** Description of studies included in the analysis

	Country	N	Type of study	Setting	Facility delivery rate	% LBW	Method of gestational age measurement
**Asia**	India (2000)[25]	12,936	RCT of newborn Vitamin A supplementation	Rural	63	33	LMP
	
	Nepal (1999)[26]	4,130	Cluster RCT of multiple micronutrient supplementation	Rural	6	39	LMP
	
	Nepal (2003)[28]	1,106	RCT of antenatal micronutrient supplementation	Peri-urban	53	22	Ultrasound
	
	Nepal (2004)[27]	23,662	Cluster RCT of newborn skin-umbilical cord cleansing with chlorhexidine	Rural	10	30	LMP
	
	Philippines (1983)[29]	3,080	Longitudinal Health-nutritional survey of infant feeding patterns	Urban	34	11	LMP, Ballard
	
	Thai (2001)[30]	4,245	Prospective follow-up of birth cohort	Urban	99	8	Best obstetric estimate (LMP, ultrasound or neonatal assessment)

**Africa**	Burkina Faso (2004)[31]	1,373	RCT of multiple micronutrient supplementation	Rural	77	17	Ultrasound at recruitment
	
	Burkina Faso (2006)[34]	1,316	RCT of maternal fortified food supplementation	Rural	84	16	Ultrasound at recruitment
	
	Tanzania (2001)[32]	7,752	RCT of maternal multiple micronutrient supplementation	Urban	98	10	LMP
	
	Zimbabwe (1997)[33,35]	14,110	RCT of postpartum maternal and neonatal Vitamin A supplementation	Urban	88	14	Capurro

**Americas**	Brazil (1982)[21]	5,914	Prospective cohort study	Urban	100	7	LMP
	
	Brazil (1993)[22]	5,279	Prospective cohort study	Urban	100	9	LMP, Dubowitz
	
	Brazil (2004)[23]	4,287	Prospective cohort study	Urban	100	11	LMP, Dubowitz, ultrasound if available
	
	Peru (1995)[24]	978	RCT of maternal zinc supplementation	Urban	100	4	LMP, clinical indications

RCT = randomized controlled trial

LMP = last menstrual period

LBW = low birthweight

### Independent variable

For each dataset, nine risk categories were created, matching the three parity categories (nulliparity, parity 1-2 as reference parity, parity ≥3) and three maternal age categories (<18 years, 18-<35 years as reference age, ≥35 years) [Table 2]. These categories were chosen because DHS uses these cut-offs to identify “high-risk fertility behavior” [36,37]. We defined parity as the number of live births before the current pregnancy. The nine risk categories are mutually exclusive. Throughout the paper, we will use the denotation of “parity category/age category” (i.e. nulliparous / age <18) to indicate categories of women who belong to both the indicated parity category and age category.

**Table 2:** Parity/age categories and their median and range of prevalence across included cohort studies

	Nulliparous	Parity 1-2 (reference)	Parity ≥3
**Age <18**	Median: 7.37% Range: 0.13-12.74% N*=9	Median: 0.76% Range: 0.01-2.65% N=0	Median: 0.00 Range: 0.00-0.22% N=0

**Age 18-<35** **(reference)**	Median: 28.27% Range: 1.20-43.72% N=14	Median: 39.93% Range: 33.28-51.50% N=14	Median: 13.42% Range: 7.17-35.64% N=14

**Age ≥35**	Median: 0.08% Range: 0.00-1.54% N=0	Median: 0.71% Range: 0.00-6.30% N=2	Median: 5.52% Range: 0.17-9.19% N=7

Median and Range are described across all 14 cohort studies.

*The Ns indicated reflects how many studies out of the 14 included studies have ≥5% prevalence in that category.

Women in a risk category were compared to those in the reference category (parity 1-2/age 18-<35), creating a binary exposure variable for each risk category. The prevalence in each risk category was calculated separately by study. We only conducted analyses on a risk category if at least half of the studies (seven out of 14) reported having a prevalence of 5% or more in that category. Calculating associations for risk categories with extremely low prevalence would produce unstable estimates with wide uncertainty.

### Outcome variables

A common SGA reference distribution and preterm definition were used across all studies. We defined SGA as below the 10^th^ percentile of the U.S. 1991 reference distribution described by Alexander and colleagues [38]. We used this reference distribution, as this is the most commonly used and cited, allowing for comparability with other studies. Preterm was defined as below 37 completed weeks of gestation. The method of gestational age measurement for each study is listed in Table 1. We also created the composite outcome variables term-appropriate for-gestational-age (AGA), term-SGA, preterm-AGA, and preterm-SGA, with term-AGA as the reference. When mortality information was available, neonatal mortality was defined as death within 28 days, and infant mortality as death within 365 days. All newborns were included in the analysis examining the outcomes of preterm, neonatal, and infant mortality, even if the child did not have a weight available to examine SGA as an outcome.

### Data analysis

Datasets were first analyzed individually. Ten of the 14 cohort study datasets were available to the primary author to analyze; the remaining four datasets were analyzed by collaborators using a standardized template. Logistic regression was used to calculate the odds ratio between a risk category (inclusion criteria for risk category described in "independent variables" section) and an adverse neonatal outcome, with parity 1-2/age 18-<35 women as reference. In addition to unadjusted analysis, available socioeconomic and maternal nutritional variables were placed in the model to determine if they confounded the association [See Supplemental Table 1 in Additional file 1 for the list of covariates adjusted for during analyses]. Odds ratios, instead of relative risks, were used, due to convergence issues in adjusted analysis. The datasets were not pooled, as each dataset contained a different set of possible confounders. Not all studies reported an association for each exposure and outcome due to low prevalence in their respective studies. Once the associations were calculated at the study level, they were meta-analyzed using the metan command in Stata. Random effects models were used to address heterogeneity across studies.

We also conducted sensitivity analyses to explore the risk of more extreme cut-offs of parity and maternal age than what DHS presently defines as high risk, using the ten datasets available to the primary author. We examined the impact of higher parity and younger age by comparing the following adjusted odds ratios (aOR): 1) higher parity: parity ≥5/age 18-<35 versus parity ≥3/age 18-<35, 2) higher parity among high age women: parity ≥5/age ≥35 versus parity ≥3/age ≥35, and 3) younger age among nulliparous women: nulliparous/age <16 versus nulliparous/age <18. For all of these aORs, the reference group remained parity 1-2/age 18-<35. No analysis was conducted to examine parity 1-2/age <16 because of the small sample size across all studies in this exposure category.

We considered an alpha value of 0.05 to be statistically significant and all tests were two-sided. We used Stata 12.0 (StataCorp. 2009. College Station, TX: StataCorp LP) for analysis.

## Results

### Distribution of parity-age exposure in study populations

Table 2 presents the median and range of prevalence for each parity/age risk category across all 14 studies. The reference parity 1-2/age 18-<35 category had a median prevalence of 39.9% (range: 33.3-51.5%). Four other categories had at least seven of the 14 studies with prevalence over 5% in the respective categories. All 14 studies reported a prevalence of over 5% for nulliparous/age 18-<35 and parity ≥3/age 18-<35, with median prevalence of 28.3% and 13.4% respectively. The nulliparous/age <18 and parity ≥3/age ≥35 categories both had at least seven studies reporting over 5% prevalence. The analyses were only conducted for these four risk categories.

### Prevalence of adverse neonatal outcomes in study populations

The prevalence of adverse neonatal outcomes in each study is shown in Supplemental Table 2 in Additional file 1. SGA prevalence was generally lowest in Latin America (range: 10.8-21.1%) and highest in Asia, particularly South Asia (South Asia range: 52.3-61.5%). Preterm prevalence followed a similar regional pattern. Neonatal mortality rates were highest in South Asia (highest rate: 42 per 1000 live births in Nepal), but infant mortality rates were comparable for the South Asian and African studies, with Latin America having the lowest rates of neonatal and infant mortality.

### Associations between parity-age exposure categories and outcomes

In nearly all cases for SGA, preterm, and mortality outcomes, inclusion of socioeconomic and maternal nutrition variables did not alter the effect sizes by more than 10% (unadjusted ORs not presented). In cases where the effect size differed by more than 10%, the 95% confidence intervals (CI) overlapped.

We report the meta-analyzed associations below. The reference parity/age category is parity 1-2/age 18-<35 for all analyses.

### SGA

Nulliparous/age <18 had a statistically significant adverse association with SGA (see Table 3). Both nulliparous/age <18 (aOR: 1.80, 95% CI: 1.62-2.01) and nulliparous/age 18-<35 (aOR 1.51, 95% CI: 1.39-1.64) had increased risk of SGA. The confidence interval for these two associations overlapped slightly. The parity ≥3/age 18-<35 category did not have an adverse association. Instead, we saw a small but statistically significant protective effect against SGA (aOR: 0.92, 95% CI 0.86-0.99). The parity ≥3/age ≥35 category had no significant association.

**Table 3:** Adjusted odds ratios for adverse outcomes, by reproductive health risk factor categories

	Nulliparous / Age <18			Nulliparous / Age 18-<35			Parity ≥3 / Age 18-<35			Parity ≥3 / Age ≥35		
**Outcome**	N*	**aOR**	**95% CI**	N*	**aOR**	**95% CI**	N*	**aOR**	**95% CI**	N*	**aOR**	**95% CI**

SGA (reference: AGA)	14	1.80	1.62, 2.01	14	1.51	1.39, 1.64	14	0.92	0.86, 0.99	13	0.98	0.87, 1.09
Preterm (reference: term)	14	1.52	1.40, 1.66	14	1.09	0.99, 1.21	14	1.20	1.06, 1.35	12	1.43	1.21, 1.69
Term-SGA (reference: term-AGA)	14	1.81	1.51, 2.16	14	1.64	1.46, 1.85	14	0.88	0.81, 0.96	13	1.06	0.93, 1.20
Preterm-AGA (reference: term-AGA)	13	1.75	1.56, 1.98	13	1.30	1.09, 1.54	13	1.13	0.98, 1.30	12	1.39	1.16, 1.65
Preterm-SGA (reference: term-AGA)	11	3.14	2.18, 4.53	14	2.67	1.97, 3.61	13	1.07	0.83, 1.38	12	1.24	1.06, 1.44
Neonatal Mortality	12	2.07	1.69, 2.54	13	1.28	1.07, 1.51	12	1.30	1.11, 1.51	10	1.66	1.23, 2.23
Infant Mortality	8	1.49	1.13, 1.97	8	1.11	0.82, 1.52	8	1.40	1.04, 1.89	8	1.36	0.92, 2.03

*N=number of studies included in the meta-analysis

Controlled for socioeconomic and maternal nutritional variables from Supplemental Table 1 in Additional file 1.

Reference exposure: parity 1-2 / Age 18-<35.

SGA = small-for-gestational-age, defined as below the 10th percentile of the U.S. 1991 reference distribution described by Alexander and colleagues [38]. AGA = appropriate-for-gestational-age. Preterm = below 37 completed weeks of gestation.

### Preterm

Almost all exposure categories had statistically significant associations with preterm birth. The nulliparous/age <18 category had the highest risk of preterm birth (aOR: 1.52, 95% CI 1.40-1.66), followed by parity ≥3/age ≥35 (aOR 1.43, 95% CI: 1.21-1.69) and parity ≥3/age 18-<35 (aOR: 1.20, 95% CI: 1.06-1.35) (see Table 3).

### Gestational age-SGA combined categories

**Figure 1:**
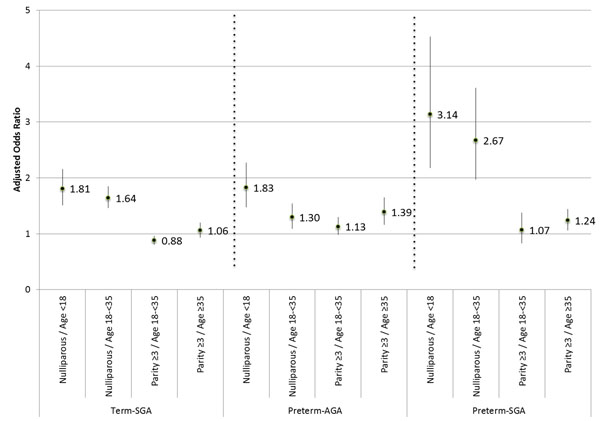
**Adjusted odds ratios for adverse outcomes, by Term-SGA, Preterm-AGA, and Preterm-SGA (reference: Term-AGA).** SGA = small-for-gestational-age, defined as below the 10^th^ percentile of the U.S. 1991 reference distribution described by Alexander and colleagues [38]. AGA = appropriate-for-gestational-age. Preterm = below 37 completed weeks of gestation.

*Term-SGA*. Nulliparous/age <18 mothers had a significant association with term-SGA (aOR: 1.81, 95% CI: 1.51-2.16). Nulliparous/age 18-<35 had slightly weaker but significant associations. The parity ≥3/age 18-<35 women had a significant protective association (aOR: 0.88, 95% CI: 0.81-0.96), and parity ≥3/age ≥35 had no association. *Preterm-AGA*. Nulliparous/age <18 had the largest association (aOR: 1.83, 95% CI: 1.48-2.27). Nulliparous/age 18-<35 and parity ≥3/age ≥35 had 30-40% significant increase in odds, and parity ≥3/age 18-<35 had no significant association. *Preterm-SGA.* Nulliparous/age <18 mothers once again had the highest association (aOR: 3.14, 95% CI: 2.18-4.53), followed by nulliparous/age 18-<35 (aOR: 2.67, 95% CI: 1.97-3.61). Parity ≥3/age ≥35 had slightly increased odds (aOR: 1.24, 95% CI: 1.06-1.44), and parity ≥3/age 18-<35 had no association. (See Table 3 and Figure 1). Relative risks (RR) were requested for use in *LiST*, and the unadjusted RRs can be found in Supplemental Table 3 in Additional file 1.

### Neonatal and infant mortality

All datasets contributed systematic neonatal mortality data except Peru (very low incidence). All risk categories had statistically significant increased odds of neonatal mortality, but the magnitude of the effect size varied. Nulliparous/age <18 had an approximately two-fold increased risk (aOR: 2.07, 95% CI 1.69-2.54). Nulliparous/age 18-<35 had a 28% increase in odds (95% CI, 1.07-1.51), parity ≥3/age 18-<35 had a 30% increase in odds (95% CI: 1.11-1.51), and parity ≥3/age ≥35 had a 66% increase in odds (95% CI: 1.23-2.23). We had fewer studies contributing infant mortality, as five of the datasets did not follow children systematically up to one year. For infant mortality, the statistically significant association only remained for nulliparous/age <18 (aOR 1.49, 95% CI 1.13-1.97) and parity ≥3/age 18-<35 (aOR 1.40, 95% CI 1.04-1.89) (See Table 3).

### Examining higher parity and lower age cut-offs

Higher parity and lower age cut-offs were examined for the ten datasets available to the primary author. When increasing the parity cut-off to 5, we did not notice large differences in odds ratios. For both parity ≥5/age 18-<35 and parity ≥5/age ≥35, the magnitude of all the associations were very similar to parity cut-off of 3. For nulliparous/age <16, we saw a consistent increase in odds ratios compared to nulliparous/age <18, particularly for preterm outcomes. However, none of the associations were statistically significantly different between the two exposure categories (Supplemental Table 4a-c in Additional file 1).

## Discussion

Our analyses found associations with adverse neonatal outcomes for some reproductive characteristics previously considered high risk, while not for others. Nulliparous women had significant associations with adverse outcomes, but particularly when mothers were also of young age; women who were both nulliparous and age <18 consistently experienced the highest risk. Young age appeared to drive preterm risk, as seen in the statistically significantly different preterm associations comparing nulliparous women age <18 to age 18-<35. When we conducted sensitivity analyses using a lower age cut-off of 16, the associations increased in magnitude, particularly for preterm outcomes. Although the change in associations were not statistically significant, this may be driven by sample size, as we had a very low prevalence of women under age 16. Several studies have reported increased rates of preterm delivery and/or neonatal mortality among young mothers [3,5,39,40]. A plausible biological explanation may be incomplete maternal physical growth and relative malnutrition, which is related to the mother’s gynecological age rather than chronological age [3]. In a U.S. study, growing adolescents accrued more fat and more weight during their pregnancy, but their infants weighed less at birth and their mothers retained more weight postpartum [41]. In resource-constrained settings, adolescent mothers may have an even larger nutritional burden; a study in rural Nepal observed lighter newborns and a larger loss of mid-upper arm circumference in pregnancy among adolescent mothers than their older counterparts [42]. The association with young age may also be related to women taking two years post-menarche or longer to reach their adult stature and pelvic dimensions [43]. We did not have data on gynecological age to explore this issue.

For age ≥35, we witnessed an increase in risk of preterm birth, but no association with SGA. The preterm association may be attributed to greater incidence of chromosomal or congenital abnormalities, or confounding by maternal morbidities such as gestational diabetes, pre-eclampsia, and hypertension, as well as chronic disease, which may be more common among older mothers [9,44]. Previous studies link advanced maternal age to increased risk of preterm delivery and poor perinatal outcomes, with age having a dose response relationship to risk [9,45]. There were not enough women in our data with advanced age to explore a possible dose response relationship of age beyond 35.

Our analysis yielded mixed results on the association between parity ≥3 and adverse outcomes. We saw no adverse association with SGA, and a weak association with preterm. WWhen we conducted sensitivity analyses by raising the parity cut-off to ≥5, we saw no dose response relationship, which puts into question an actual biological association between high parity and adverse newborn outcomes. This hypothesis is supported by some previous literature. A separate analysis in this supplement using DHS datasets [46] noted that the increased mortality risk among high parity infants was attributable mostly, if not entirely, to confounders strongly correlated with the mother having high fertility at the end of her reproductive period. A series of studies from Israel also attributed higher rates of negative outcomes to socioeconomic and environmental factors highly correlated with high parity [10,47,48]. Finally, several studies have reported that grand multiparous women had no increased risk in contexts where women were economically stable or had proper access to care [49-51]. In light of these findings, we believe that residual confounding, and not biological mechanisms, may fully account for the association between high parity and adverse outcomes. It is unclear why we see a slight, but significant protective association between parity ≥3 and SGA. With the parity ≥5 cut-off, the magnitude of the associations remained almost identical, although the associations lost statistical significance. The lack of change in risk with increasing parity may again hint that confounders not captured by the covariates in our data are at play.

All risk categories had a statistically significant association with neonatal mortality, with age <18 and age ≥35 appearing to be the largest drivers of mortality. Only nulliparous/age <18 and parity≥3/age 18-<35 retained statistically significant associations with infant mortality. The parity and age risks may be operating on mortality through SGA/preterm and/or confounders like socioeconomic characteristics or access to care that may not have been fully captured by the available control variables.

An increase in contraceptive prevalence has been effective in reducing high parity and advanced age births, but not in delaying maternal age at first birth. Stover and Ross report an extremely low correlation of R^2^=0.05 between contraceptive prevalence rate and percentage of births with mothers age <18 years [52]. This implies the need to focus on addressing adolescent pregnancies and for innovative strategies that may be more successful in delaying age at first birth. This may mean expanding the scope of interventions beyond the health sector; for instance, some literature suggests that educational attainment delays age to first birth [53-55]. In finding effective interventions, it is also important to understand factors beyond knowledge and access that motivate reproductive decisions. For instance, survival of previous children and desired family size could be driving higher age and parity births, and depending on the circumstances, ensuring child survival may be more effective in addressing reproductive risk than family planning. Finally, there is a need to address the equity issues surrounding access to family planning. There are substantial gaps in contraceptive prevalence between women belonging to high wealth quintiles and to low wealth quintiles [56,57]. Those who have the least access are most likely the ones who need it the most, as suggested by our concerns with residual confounding.

The strength of our analyses is the use of high quality, population-based cohort data that allows us to make better inferences of causation. We have examined how parity and maternal age together may impact outcome, rather than simply using one or the other as a control variable. We were also able to use standardized definitions for both exposures and outcomes, enhancing comparability across studies. Previous studies have used a variety of cut-offs and definitions for parity and age [3,4,15,16]. Taking SGA as an example, even using the most widely accepted definition (under 10^th^ percentile of a reference distribution), the prevalence of SGA can vary depending on which reference distribution is being used. SGA prevalence ranged from 5-72% when applying existing reference distributions to a single population sample (Joanne Katz, personal communication).

A weakness of our study is the limit of confounders available in each primary study. We controlled for the available confounders, but there still remains the possibility of residual confounding. In addition to our aforementioned discussion on high parity and residual confounding, there is also literature suggesting that controlling for socioeconomic characteristics largely attenuates the risk of adverse outcomes with young maternal age [58,59]. Although there is strong evidence for biological mechanisms linking nulliparity to adverse outcomes, those associations may also still be partially confounded by factors like quality and/or access to health care. Birth interval is another reproductive factor that could be a confounder, but was not included in this analysis, as a majority of the studies did not have that information. A separate study in this supplement evaluates the associations between birth intervals and poor outcomes, controlling for parity and maternal age in a subset of our datasets with relevant information [60]. Variables like survival of previous children may reflect underlying maternal health and socioeconomic conditions that are not well captured in the available variables.

Due to limitations of the data, we were not able to explore gravidity (all pregnancies), instead of parity (all live births), as the exposure. Using gravidity instead may have given us additional insight into the mechanisms linking parity and birth outcome; particularly with stillbirths and less so with miscarriages, we would expect mothers to endure similar nutritional demand during pregnancy as a live birth. Furthermore, by not taking into account miscarriages and stillbirths as outcomes, we may be underestimating the negative impact of some of the risk factors. There is existing literature that links nulliparity with stillbirth and intrapartum-related neonatal mortality [6,61,62]. A systematic review showed increased risk of stillbirth and perinatal death with older maternal age [63]. In contrast, publications have produced mixed findings pertaining to the associations between high parity and stillbirth [6,64] as well as young maternal age and stillbirth [3]. Collecting more detailed information may help us better understand the mechanisms linking reproductive health risk factors to adverse fetal and perinatal outcomes, and support better-designed and better-timed interventions for at-risk mothers and newborns.

Although the studies included in our analyses are all population-based studies, we acknowledge that the data are not nationally representative. However, we believe that the risk associations we present here are less likely to be influenced by this, as prevalence estimates may be. Finally, we were only able to conduct sensitivity analyses of the parity ≥5 and age <16 years cut-offs on the datasets available to the primary author and with high enough prevalence in the respective categories.

## Conclusions

The highest odds of adverse neonatal outcomes associated with nulliparous/age <18 mothers and also the increased odds seen among parity ≥3/age ≥35 mothers indicate that reproductive health interventions and education need to occur throughout a woman’s reproductive period. Delaying maternal age at first birth could decrease risk for all adverse neonatal outcomes, but family planning interventions appear to be the least successful in addressing this risk factor. This calls upon family planning experts to refocus their efforts on finding interventions that will more effectively address this issue.

For *LiST*, we recommend including no association between high parity and adverse newborn outcomes. We recommend including the associations presented here for nulliparity, age <18 years, and age ≥35 years, with the caveat that the associations may still be influenced by residual confounding.

## Competing interests

We report no competing interests.

## Authors’ contributions

NK was responsible for study design, data collection, literature review, analyses of primary datasets and the meta-analyses, and writing of the manuscript. ACL and JK were responsible for study design, data collection, interpretation of results, and helped draft the manuscript.

MFS, AS, JPV helped analyze primary datasets and reviewed the manuscript. FB, LC, PC, WF, JH, LH, AM, RN, DO, DR, JT, and AV contributed data and reviewed the manuscript. REB contributed to study design and interpretation of results, and reviewed the manuscript. All authors read and approved the final manuscript.

## Supplementary Material

Additional file 1 Supplemental material. Click here for file

## Declarations

The publication costs for this supplement were funded by a grant from the Bill & Melinda Gates Foundation to the US Fund for UNICEF (grant 43386 to "Promote evidence-based decision making in designing maternal, neonatal, and child health interventions in low- and middle-income countries”). The Supplement Editor is the principle investigator and lead in the development of the Lives Saved Tool (LiST), supported by grant 43386. He declares that he has no competing interests.

This article has been published as part of *BMC Public Health* Volume 13 Supplement 3, 2013: The Lives Saved Tool in 2013: new capabilities and applications. The full contents of the supplement are available online at http://www.biomedcentral.com/bmcpublichealth/supplements/13/S3.

## References

[B1] Kiely JL Paneth N Susser M An assessment of the effects of maternal age and parity in different components of perinatal mortality American Journal of Epidemiology 1986 123 3 444 454 3946390 10.1093/oxfordjournals.aje.a114259

[B2] Usta IM Zoorob D Abu-Musa A Naassan G Nassar AH Obstetric outcome of teenage pregnancies compared with adult pregnancies Acta Obstetricia et Gynecologica Scandinavica 2008 87 2 178 183 10.1080/00016340701803282 18231885

[B3] Gibbs CM Wendt A Peters S Hogue CJ The impact of early age at first childbirth on maternal and infant health Paediatric and Perinatal Epidemiology 2012 26 Suppl 1 259 284 22742615 10.1111/j.1365-3016.2012.01290.x PMC4562289

[B4] Shah PS Parity and low birth weight and preterm birth: a systematic review and meta-analyses Acta Obstetricia et Gynecologica Scandinavica 2010 89 7 862 875 10.3109/00016349.2010.486827 20583931

[B5] Scholl TO Hediger ML Huang J Johnson FE Smith W Ances IG Young maternal age and parity. Influences on pregnancy outcome Annals of Epidemiology 1992 2 5 565 575 10.1016/1047-2797(92)90001-7 1342308

[B6] Lee AC Mullany LC Tielsch JM Katz J Khatry SK LeClerq SC Adhikari RK Darmstadt GL Community-based stillbirth rates and risk factors in rural Sarlahi, Nepal Int J Gynecol Obstet 2011 113 3 199 204 10.1016/j.ijgo.2010.12.015 21458812

[B7] Kramer KL Lancaster JB Teen motherhood in cross-cultural perspective Annals of Human Biology 2010 37 5 613 628 10.3109/03014460903563434 20205610

[B8] Carolan M Frankowska D Advanced maternal age and adverse perinatal outcome: a review of the evidence Midwifery 2011 27 6 793 801 10.1016/j.midw.2010.07.006 20888095

[B9] Yogev Y Melamed N Bardin R Tenenbaum-Gavish K Ben-Shitrit G Ben-Haroush A Pregnancy outcome at extremely advanced maternal age Am J Obstet Gynecol 2010 203 6 558e.1 558-e.7 20965486 10.1016/j.ajog.2010.07.039

[B10] Seidman DS Gale R Slater PE Ever-Hadani P Harlap S Does grand multiparity affect fetal outcome? Int J Gynaecol Obstet 1987 25 1 1 7 10.1016/0020-7292(87)90176-7 2883039

[B11] Sharma V Katz J Mullany LC Khatry SK LeClerq SC Shrestha SR Darmstadt GL Tielsch JM Young maternal age and the risk of neonatal mortality in rural Nepal Archives of Pediatrics & Adolescent Medicine 2008 162 9 828 835 10.1001/archpedi.162.9.828 18762599 PMC2535853

[B12] Taffa N A comparison of pregnancy and child health outcomes between teenage and adult mothers in the slums of Nairobi, Kenya International Journal of Adolescent Medicine and Health 2003 15 4 321 329 14719414 10.1515/ijamh.2003.15.4.321

[B13] Finlay JE Ozaltin E Canning D The association of maternal age with infant mortality, child anthropometric failure, diarrhoea and anaemia for first births: evidence from 55 low- and middle-income countries BMJ Open 2011 1 2 e000226 10.1136/bmjopen-2011-000226 22021886 PMC3191600

[B14] Titaley CR Dibley MJ Agho K Roberts CL Hall J Determinants of neonatal mortality in Indonesia BMC Public Health 2008 8 232 10.1186/1471-2458-8-232 18613953 PMC2478684

[B15] National Statistical Office (Malawi) ORC Macro Malawi Demographic and Health Survey 2004 2005 Claverton, Maryland

[B16] Grisaru-Granovsky S Gordon ES Haklai Z Samueloff A Schimmel MM Effect of interpregnancy interval on adverse perinatal outcomes--a national study Contraception 2009 80 6 512 518 10.1016/j.contraception.2009.06.006 19913144

[B17] Allen SJ Raiko A O'Donnell A Alexander ND Clegg JB Causes of preterm delivery and intrauterine growth retardation in a malaria endemic region of Papua New Guinea Archives of Disease in Childhood: Fetal and Neonatal Edition 1998 79 2 F135 140 10.1136/fn.79.2.F135 9828741 PMC1720830

[B18] Clausson B Cnattingius S Axelsson O Preterm and term births of small for gestational age infants: a population-based study of risk factors among nulliparous women British Journal of Obstetrics and Gynaecology 1998 105 9 1011 1017 10.1111/j.1471-0528.1998.tb10266.x 9763054

[B19] Sachdev HS Hall A Walker N Development and use of the Lives Saved Tool (LiST): A model to estimate the impact of scaling up proven interventions on maternal, neonatal, and child mortality International Journal of Epidemiology 2010 i5 i205 10.1093/ije/dyq173 PMC3066427 21036879

[B20] Katz J Lee AC Kozuki N Lawn J Cousens S Blencowe H Ezzati M Mortality risk in preterm and small-for-gestational-age infants in low-income and middle-income countries: a pooled country analysis Lancet 2013 382 9890 417 425 10.1016/S0140-6736(13)60993-9 23746775 PMC3796350

[B21] Victora CG Barros FC Cohort profile: the 1982 Pelotas (Brazil) birth cohort study International Journal of Epidemiology 2006 35 2 237 242 16373375 10.1093/ije/dyi290

[B22] Victora CG Hallal PC Araujo CL Menezes AM Wells JC Barros FC Cohort profile: the 1993 Pelotas (Brazil) birth cohort study International Journal of Epidemiology 2008 37 4 704 709 17846051 10.1093/ije/dym177

[B23] Santos IS Barros AJ Matijasevich A Domingues MR Barros FC Victora CG Cohort profile: the 2004 Pelotas (Brazil) birth cohort study International Journal of Epidemiology 2011 40 6 1461 1468 10.1093/ije/dyq130 20702597 PMC3235016

[B24] Caulfield LE Zavaleta N Figueroa A Leon Z Maternal zinc supplementation does not affect size at birth or pregnancy duration in Peru The Journal of Nutrition 1999 129 8 1563 1568 10419991 10.1093/jn/129.8.1563

[B25] Rahmathullah L Tielsch JM Thulasiraj RD Katz J Coles C Devi S John R Prakash K Sadanand AV Edwin N Impact of supplementing newborn infants with vitamin A on early infant mortality: community based randomised trial in southern India BMJ 2003 327 7409 254 10.1136/bmj.327.7409.254 12896935 PMC167159

[B26] Christian P Khatry SK Katz J Pradhan EK LeClerq SC Shrestha SR Adhikari RK Sommer A West KP Jr. Effects of alternative maternal micronutrient supplements on low birth weight in rural Nepal: double blind randomised community trial BMJ 2003 326 7389 571 10.1136/bmj.326.7389.571 12637400 PMC151518

[B27] Tielsch JM Darmstadt GL Mullany LC Khatry SK Katz J LeClerq SC Shrestha S Adhikari R Impact of newborn skin-cleansing with chlorhexidine on neonatal mortality in southern Nepal: a community-based, cluster-randomized trial Pediatrics 2007 119 2 e330 340 10.1542/peds.2006-1192 17210728 PMC2364722

[B28] Osrin D Vaidya A Shrestha Y Baniya RB Manandhar DS Adhikari RK Filteau S Tomkins A Costello AM Effects of antenatal multiple micronutrient supplementation on birthweight and gestational duration in Nepal: double-blind, randomised controlled trial Lancet 2005 365 9463 955 962 10.1016/S0140-6736(05)71084-9 15766997

[B29] Adair LS Low birth weight and intrauterine growth retardation in Filipino infants Pediatrics 1989 84 4 613 622 2780122

[B30] Isaranurug S Mo-suwan L Choprapawon C A population-based cohort study of effect of maternal risk factors on low birthweight in Thailand Journal of the Medical Association of Thailand = Chotmaihet thangphaet 2007 90 12 2559 2564 18386704

[B31] Roberfroid D Huybregts L Lanou H Henry MC Meda N Menten J Kolsteren P Effects of maternal multiple micronutrient supplementation on fetal growth: a double-blind randomized controlled trial in rural Burkina Faso Am J Clin Nutr 2008 88 5 1330 1340 18996870 10.3945/ajcn.2008.26296

[B32] Fawzi WW Msamanga GI Urassa W Hertzmark E Petraro P Willett WC Spiegelman D Vitamins and perinatal outcomes among HIV-negative women in Tanzania The New England Journal of Medicine 2007 356 14 1423 1431 10.1056/NEJMoa064868 17409323

[B33] Malaba LC Iliff PJ Nathoo KJ Marinda E Moulton LH Zijenah LS Zvandasara P Ward BJ Humphrey JH Effect of postpartum maternal or neonatal vitamin A supplementation on infant mortality among infants born to HIV-negative mothers in Zimbabwe The American Journal of Clinical Nutrition 2005 81 2 454 460 15699235 10.1093/ajcn.81.2.454

[B34] Huybregts L Roberfroid D Lanou H Menten J Meda N Van Camp J Kolsteren P Prenatal food supplementation fortified with multiple micronutrients increases birth length: a randomized controlled trial in rural Burkina Faso The American Journal of Clinical Nutrition 2009 90 6 1593 1600 10.3945/ajcn.2009.28253 19812173

[B35] Humphrey JH Hargrove JW Malaba LC Iliff PJ Moulton LH Mutasa K Zvandasara P Nathoo KJ Mzengeza F Chidawanyika H HIV incidence among post-partum women in Zimbabwe: risk factors and the effect of vitamin A supplementation AIDS 2006 20 10 1437 1446 10.1097/01.aids.0000233578.72091.09 16791019

[B36] Ministry of Health (Guyana) GRPA ORC Macro Guyana HIV/AIDS Indicator Survey 2005 2006 Calverton

[B37] El-Zanaty F Way A Ministry of Health, El-Zanaty and Associates, and Macro International Egypt Demographic and Health Survey 2008 2009 Cairo

[B38] Alexander GR Himes JH Kaufman RB Mor J Kogan M A United States national reference for fetal growth Obstetrics and Gynecology 1996 87 2 163 168 10.1016/0029-7844(95)00386-X 8559516

[B39] Conde-Agudelo A Belizan JM Lammers C Maternal-perinatal morbidity and mortality associated with adolescent pregnancy in Latin America: Cross-sectional study American Journal of Obstetrics and Gynecology 2005 192 2 342 349 10.1016/j.ajog.2004.10.593 15695970

[B40] Chen XK Wen SW Fleming N Demissie K Rhoads GG Walker M Teenage pregnancy and adverse birth outcomes: a large population based retrospective cohort study International Journal of Epidemiology 2007 36 2 368 373 10.1093/ije/dyl284 17213208

[B41] Scholl TO Hediger ML Schall JI Khoo CS Fischer RL Maternal growth during pregnancy and the competition for nutrients The American Journal of Clinical Nutrition 1994 60 2 183 188 8030595 10.1093/ajcn/60.2.183

[B42] Katz J Khatry SK LeClerq SC West KP Christian P The post-partum mid-upper arm circumference of adolescents is reduced by pregnancy in rural Nepal Maternal & Child Nutrition 2010 6 3 287 295 20929500 10.1111/j.1740-8709.2009.00211.x PMC2953737

[B43] Moerman ML Growth of the birth canal in adolescent girls American Journal of Obstetrics and Gynecology 1982 143 5 528 532 7091223 10.1016/0002-9378(82)90542-7

[B44] Weerasekera DS Udugama SG Pregnancy at 40 and over: a case-control study in a developing country J Obstet Gynaecol 2003 23 6 625 627 10.1080/01443610310001604385 14617463

[B45] Cleary-Goldman J Malone FD Vidaver J Ball RH Nyberg DA Comstock CH Saade GR Eddleman KA Klugman S Dugoff L Impact of maternal age on obstetric outcome Obstetrics and Gynecology 2005 105 5 Pt 1 983 990 15863534 10.1097/01.AOG.0000158118.75532.51

[B46] Kozuki N Sonneveldt E Walker N Residual confounding explains the association between high parity and child mortality BMC Public Health 2013 13 Suppl 3 S5 24564642 10.1186/1471-2458-13-S3-S5 PMC3847621

[B47] Mor-Yosef S Seidman DS Samueloff A Schenker JG The effects of the socioeconomic status on the perinatal outcome of grand multipara European Journal of Obstetrics, Gynecology, and Reproductive Biology 1990 36 1-2 117 123 10.1016/0028-2243(90)90057-8 2365117

[B48] Seidman DS Dollberg S Stevenson DK Gale R The effects of high parity and socioeconomic status on obstetric and neonatal outcome Archives of Gynecology and Obstetrics 1991 249 3 119 127 10.1007/BF02391578 1772264

[B49] Kumari AS Badrinath P Extreme grandmultiparity: is it an obstetric risk factor? European Journal of Obstetrics, Gynecology, and Reproductive Biology 2002 101 1 22 25 10.1016/S0301-2115(01)00498-5 11803095

[B50] Eidelman AI Kamar R Schimmel MS Bar-On E The grandmultipara: is she still a risk? American Journal of Obstetrics and Gynecology 1988 158 2 389 392 3341414 10.1016/0002-9378(88)90161-5

[B51] Hughes PF Morrison J Grandmultiparity--not to be feared? An analysis of grandmultiparous women receiving modern antenatal care Int J Gynaecol Obstet 1994 44 3 211 217 10.1016/0020-7292(94)90168-6 7909758

[B52] Stover J Ross J Changes in the distribution of high-risk births associated with changes in contraceptive prevalence BMC Public Health 2013 13 Suppl 3 S4 24564577 10.1186/1471-2458-13-S3-S4 PMC3847521

[B53] Grindstaff BT C Dewit D Educational attainment, age at first birth and lifetime fertility: an analysis of Canadian fertility survey data Canadian Review of Sociology 1991 28 3 324 339

[B54] Ferre C Bank TW Age at First Child - Does Education Delay Fertility Timing? Policy Research Working Paper 2009

[B55] DePaoli A Centro Studi Luca D'Agliano Education, Teenage Fertility and Labour Market Participation, Evidence from Ecuador Development Studies Working Papers 2011

[B56] World Bank Reproductive Health at a Glance: Burkina Faso 2011 Washington DC

[B57] World Bank Reproductive Health at a Glance: Mozambique 2011 Washington, DC

[B58] Markovitz BP Cook R Flick LH Leet TL Socioeconomic factors and adolescent pregnancy outcomes: distinctions between neonatal and post-neonatal deaths? BMC Public Health 2005 5 79 10.1186/1471-2458-5-79 16042801 PMC1190191

[B59] Satin AJ Leveno KJ Sherman ML Reedy NJ Lowe TW McIntire DD Maternal youth and pregnancy outcomes: middle school versus high school age groups compared with women beyond the teen years American Journal of Obstetrics and Gynecology 1994 171 1 184 187 10.1016/0002-9378(94)90467-7 8030697

[B60] Kozuki N Lee AC Silveria M Victora CG Adair L Humphrey J Ntozini R Black RE Katz J The associations of birth intervals with small-for-gestational-age, preterm, and neonatal and infant mortality: a meta-analysis BMC Public Health 2013 13 Suppl 3 S3 24564484 10.1186/1471-2458-13-S3-S3 PMC3847557

[B61] Fretts R Stillbirth epidemiology, risk factors, and opportunities for stillbirth prevention Clinical Obstetrics and Gynecology 2010 53 3 588 596 10.1097/GRF.0b013e3181eb63fc 20661043

[B62] Kupka R Kassaye T Saathoff E Hertzmark E Msamanga GI Fawzi WW Predictors of stillbirth among HIV-infected Tanzanian women Acta Obstetricia et Gynecologica Scandinavica 2009 88 5 584 592 10.1080/00016340902835901 19306132 PMC2796303

[B63] Huang L Sauve R Birkett N Fergusson D van Walraven C Maternal age and risk of stillbirth: a systematic review CMAJ 2008 178 2 165 172 18195290 10.1503/cmaj.070150 PMC2175002

[B64] Chalumeau M Bouvier-Colle MH Breart G Can clinical risk factors for late stillbirth in West Africa be detected during antenatal care or only during labour? International Journal of Epidemiology 2002 31 3 661 668 10.1093/ije/31.3.661 12055171

