# Supplementary material for: The associations of parity and maternal age with small-for-gestational-age, preterm, and neonatal and infant mortality: a meta-analysis
Source: BMC Public Health. 2013 Sep 17;13(Suppl 3):S2. doi: 10.1186/1471-2458-13-S3-S2 (PMC3847520; doi:10.1186/1471-2458-13-S3-S2)
Supplement: Additional file 1 — Supplemental material. [file 1471-2458-13-S3-S2-S1.pdf]

Kozuki N, et al. The Associations of Parity and Maternal Age with Small-for-Gestational-Age, Preterm, and Neonatal and Infant Mortality: A Meta-analysis

Supplemental Table 1: Control variables used for each study

	Study	Socioeconomic	Maternal nutrition
**Asia**	**India (2000)[25]**	Maternal education, land ownership, availability of electricity	Not available
	**Nepal (1999)[26]**	Maternal education, ethnicity, and land ownership	Height, Weight, MUAC
	**Nepal (2003)[28]**	Maternal education, land ownership	Height, weight
	**Nepal (2004)[27]**	Maternal education, ethnicity, land ownership	Not available
	**Philippines (1983)[29]**	Land ownership, housing structure	Height, MUAC, weight (at 6-7 months gestation)
	**Thai (2001)[30]**	Income quintiles	Height, weight
**Africa**	**Burkina Faso (2004)[31]**	Maternal education, ethnicity	Height, MUAC
	**Burkina Faso (2006)[34]**	Maternal education, ethnicity	Height, MUAC
	**Tanzania (2001)[32]**	ANC visits, education	Height
	**Zimbabwe (1997)[33, 35]**	Maternal education	Postpartum MUAC
**America**	**Brazil (1982)[21]**	Income, maternal education	Height, weight
	**Brazil (1993)[22]**	Income, maternal education	Height, weight
	**Brazil (2004)[23]**	Income, maternal education	Height, weight
	**Peru (1995)[24]**	Maternal education, housing structure	Height, Weight, MUAC

Supplemental Table 2: Prevalence of adverse outcomes, by study

Region	Study	SGA	Preterm	Term-AGA	Term-SGA	Preterm-AGA	Preterm-SGA	Neonatal Mortality Rate**	Infant Mortality Rate**
**Asia**	**India (2000)[25]**	61.5	13.3	28.4	58.6	10.2	2.8	33	N/A
	**Nepal (1999)[26]**	55.7	22.4	28.1	50.7	16.3	4.9	42	91
	**Nepal (2003)[28]**	52.5	9.3	43.3	49.5	4.3	3.0	26	N/A
	**Nepal (2004)[27]**	52.3	17.7	34.4	47.8	13.3	4.5	32	N/A
	**Philippines (1983)[29]**	25.3	17.0	60.3	22.7	14.4	2.6	13	33
	**Thai (2001)[30]**	22.2	9.1	69.8	21.2	8.1	1.0	3	6
**Africa**	**Burkina Faso (2004)[31]**	34.9	16.2	54.1	32.5	11.1	2.4	18	62
	**Burkina Faso (2006)[34]**	29.1	17.7	59.2	26.3	11.7	2.7	19	N/A
	**Tanzania (2001)[32]**	19.7	16.7	64.7	15.6	18.6	1.1	27	N/A
	**Zimbabwe (1997)[33, 35]**	32.8	7.6	62.6	29.9	4.7	2.9	9*	78
**America**	**Brazil (1982)[21]**	21.1	5.0	61.7	12.4	3.9	1.0	14	28
	**Brazil (1993)[22]**	20.4	10.2	63.4	15.4	9.23	1.0	8	14
	**Brazil (2004)[23]**	16.7	16.1	63.1	11.8	14.3	1.7	12	17
	**Peru (1995)[24]**	10.8	5.3	84.6	10.1	4.7	0.6	N/A	N/A

Prevalences are calculated for the full study cohort.
*Enrollment of newborns occurred up to 96 hours after birth, and the study may have missed neonatal deaths prior to enrollment.
** Per 1000 live birth
SGA = small-for-gestational-age, defined as below the 10th percentile of the U.S. 1991 reference distribution described by Alexander and colleagues [38]. AGA = appropriate-for-gestational-age. Preterm = below 37 completed weeks of gestation

Kozuki N, et al. The Associations of Parity and Maternal Age with Small-for-Gestational-Age, Preterm, and Neonatal and Infant Mortality: A Meta-analysis

Supplemental Table 3: Unadjusted relative risk for preterm-SGA combinations, by reproductive health risk factor categories

	Nulliparous / Age <18			Nulliparous / Age 18-<35			Parity ≥3 / Age 18-<35			Parity ≥3 / Age ≥35		
**Outcome**	**N***	**RR**	**95% CI**	**N***	**RR**	**95% CI**	**N***	**RR**	**95% CI**	**N***	**RR**	**95% CI**
Term-SGA	14	1.47	1.36, 1.60	14	1.25	1.19, 1.31	14	0.99	0.95, 1.02	13	1.05	0.98, 1.13
Preterm-AGA	14	1.74	1.52, 2.00	14	1.21	1.06, 1.37	14	1.12	0.99, 1.27	12	1.33	1.15, 1.54
Preterm-SGA	11	2.74	2.12, 3.55	14	1.73	1.42, 2.12	13	1.13	1.00, 1.27	12	1.57	1.29, 1.91

*N = Number of studies included in the meta-analysis
SGA = small-for-gestational-age, defined as below the 10th percentile of the U.S. 1991 reference distribution described by Alexander and colleagues [38]. AGA = appropriate-for-gestational-age. Preterm = below 37 completed weeks of gestation
Reference outcome = Term-AGA
Reference exposure = parity 1-2 / Age 18-<35

Supplemental Table 4a: Comparison of adjusted odds ratios of Parity ≥5 / Age 18-<35 and Parity ≥3 / Age 18-<35 as exposures

	Parity ≥5 / Age 18-<35			Parity ≥3 / Age 18-<35		
**Outcome**	**N**	**aOR**	**95% CI**	**N**	**aOR**	**95% CI**
SGA (reference: AGA)	10	0.91	0.76, 1.09	14	0.92	0.86, 0.99
Preterm (reference: Term)	10	1.23	0.98, 1.54	14	1.20	1.06, 1.35
Term-SGA (reference: Term-AGA)	10	0.89	0.77, 1.03	14	0.88	0.81, 0.96
Preterm-AGA (reference: Term-AGA)	9	1.09	0.82, 1.44	14	1.13	0.98, 1.30
Preterm-SGA (reference: Term-AGA)	8	1.42	1.01, 2.00	13	1.07	0.83, 1.38
Neonatal Mortality	6	1.17	0.92, 1.50	12	1.30	1.11, 1.51
Infant Mortality	3	1.33	0.97, 1.82	8	1.40	1.04, 1.89

Supplemental Table 4b: Comparison of adjusted odds ratios of Parity ≥5 / Age ≥35 and Parity ≥5 / Age ≥35 as exposures

	Parity ≥5 / Age ≥35			Parity ≥3 / Age ≥35		
**Outcome**	**N**	**aOR**	**95% CI**	**N**	**aOR**	**95% CI**
SGA (reference: AGA)	9	0.92	0.79, 1.07	13	0.98	0.87, 1.09
Preterm (reference: Term)	8	1.54	1.25, 1.89	12	1.43	1.21, 1.69
Term-SGA (reference: Term-AGA)	9	0.97	0.83, 1.12	13	1.06	0.93, 1.20
Preterm-AGA (reference: Term-AGA)	8	1.38	1.07, 1.77	12	1.39	1.16, 1.65
Preterm-SGA (reference: Term-AGA)	7	1.59	1.18, 2.14	12	1.24	1.06, 1.44
Neonatal Mortality	5	2.01	1.16, 3.48	10	1.66	1.23, 2.23
Infant Mortality	5	1.40	0.88, 2.23	8	1.36	0.92, 2.03

Supplemental Table 4c: Comparison of adjusted odds ratios of nulliparous / age <16 and nulliparous / age <18 as exposures

	Nulliparous / Age <16			Nulliparous / Age <18		
**Outcome**	**N**	**aOR**	**95% CI**	**N**	**aOR**	**95% CI**
SGA (reference: AGA)	9	1.87	1.33, 2.62	14	1.80	1.62, 2.01
Preterm (reference: Term)	9	1.55	1.26, 1.91	14	1.52	1.40, 1.66
Term-SGA (reference: Term-AGA)	9	2.25	1.36, 3.72	14	1.81	1.51, 2.16
Preterm-AGA (reference: Term-AGA)	8	2.56	1.82, 3.60	13	1.75	1.56, 1.98
Preterm-SGA (reference: Term-AGA)	6	5.29	3.35, 8.34	11	1.06	0.21, 5.35
Neonatal Mortality	6	3.05	1.59, 5.87	12	2.07	1.69, 2.54
Infant Mortality	4	1.66	0.62, 4.45	8	1.49	1.13, 1.97

Kozuki N, et al. The Associations of Parity and Maternal Age with Small-for-Gestational-Age, Preterm, and Neonatal and Infant Mortality: A Meta-analysis

SGA = small-for-gestational-age, defined as below the 10th percentile of the U.S. 1991 reference distribution described by Alexander and colleagues [38]. AGA = appropriate-for-gestational-age. Preterm = below 37 completed weeks of gestation.
Reference exposure: parity 1-2 / age 18-<35
N = Number of studies included in the meta-analysis
